# Short-Term Standard Diet Consumption Prior to the Oral Fat Tolerance Test Modulates the Postprandial Triglyceride Response

**DOI:** 10.3390/metabo13091019

**Published:** 2023-09-17

**Authors:** Fulya Balaban Yucesan, Eda Nur Demir, Serap Ozer Yaman, Asım Orem, Busra Dokuz Murat, Busra Bilgin

**Affiliations:** 1 Department of Medical Biochemistry, Faculty of Medicine, Karadeniz Technical University, 61080 Trabzon, Turkey; nureda-demir@hotmail.com (E.N.D.); serapozer@ktu.edu.tr (S.O.Y.); aorem64@ktu.edu.tr (A.O.); dytbusrabilgin@gmail.com (B.B.); 2 Department of Nutrition and Dietetics, Institute of Health Sciences, Marmara University, 34854 Istanbul, Turkey; dyt.busradkz@gmail.com

**Keywords:** fat load test, habitual diet, postprandial lipemic response, short-term diet, triglycerides

## Abstract

We hypothesized that the consumption of a 3-day standard diet (SD) prior to the oral fat tolerance test (OFTT), used to evaluate postprandial lipemia, may counteract the undesirable effects of individual dietary habits on the test results. The OFTT was applied to 22 healthy adults (11 females and 11 males), after their habitual diets (HDs) and following the consumption of a 3-day SD (45–60% energy from carbohydrate, 20–35% from fat, and 10–20% from protein). Plasma triglyceride (TG) concentrations were measured during fasting and at the fourth hour of the OFTT. A 3-day SD significantly reduced fasting and fourth-hour TG concentrations and delta TG values by 10%, 12.8%, and 22.7%, respectively. Decreases were observed in fasting and fourth-hour TG and delta TG values following the 3-day SD compared to the HD in subjects with fasting TG concentrations between 89 and 180 mg/dL (*p* = 0.062, *p* = 0.018, and 0.047, respectively). As a result, the consumption of a 3-day standardized diet prior to the OFTT may be useful to eliminate the false positive or negative effects of individual dietary habits on test results and to correctly identify individuals who should be administered the OFTT.

## 1. Introduction

Postprandial lipemia refers to alterations in plasma lipid profiles during the post-prandial period and is mainly characterized by an increase in triglyceride-rich lipoproteins (TRLs) and their remnants [1,2,3]. Since individuals spend more than 18 h a day in the postprandial state, research has suggested that non-fasting or postprandial triglyceride (TG) levels provide a better reflection of plasma atherogenic lipoprotein levels [4], and are superior to fasting TG in predicting the risk of cardiovascular disease (CVD) [5]. Epidemiological and case–control studies in the last decade have described non-fasting and postprandial TG concentrations as clinically significant and independent predictors of the risk of CVD [4,5,6,7,8,9,10]. The assessment of postprandial TGs by means of oral fat load testing is more controlled than unstandardized non-fasting TG evaluation [6], and suggestions have been made regarding the clinical applicability of postprandial testing [6]. The oral fat tolerance test (OFTT) was therefore developed in order to determine the postprandial TG response by measuring the plasma TG concentrations after a single standardized high-fat meal. The OFTT methodology described by an expert panel is sufficient for the evaluation in terms of postprandial hypertriglyceridemia [11,12]. According to the panel recommendations, the OFTT should consist of a single measurement of TG four hours after a test meal with a macronutrient composition of approximately 75 g of fat, 25 g of carbohydrate, and 10 g of protein after eight-hour fasting [11,12]. It has also been suggested that the OFTT can be applied to individuals with fasting TG concentrations of 89–180 mg/dL, and postprandial TG concentrations > 220 mg/dL at any time after the test are regarded as representing an abnormal test response [11,12].

In order to increase the clinical usefulness of the OFTT for the early detection of dyslipidemia, an abbreviated protocol was developed, the test meal was standardized, and the test reliability and reproducibility were investigated [13,14,15,16]. Numerous factors, such as genetic background, age, gender, diet, exercise, and insulin resistance, play important roles in the postprandial TG response and affect the OFTT results [17,18,19,20,21,22]. As noted above, the analytical phase of the OFTT is more highly standardized, while recommendations concerning the pre-analytical phase of the OFTT are limited. The effect of foods and food ingredients on the postprandial TG response was reported in a systematic review and meta-analysis [23]. Postprandial lipemia is known to be closely related to individual dietary habits [24,25], and may represent one of the important factors affecting OFTT results. Therefore, false positive or negative effects of individual dietary habits on OFTT results need to be eliminated.

We hypothesized that the consumption of a standard diet for three days prior to the OFTT may affect the standardization of individuals’ lipid metabolism and provide an improvement in the OFTT results. The purpose of this study was to determine whether a 3-day standard diet would affect the OFTT results.

## 2. Materials and Methods

### 2.1. Study Population

The study population consisted of 22 healthy adult volunteers, 11 female and 11 male, with a mean age of 33 ± 5 years (range 26 to 43). Since age and gender are important factors affecting the postprandial TG response, particular attention was paid to age and gender distributions in the study population. The health status of the volunteers was evaluated by means of anamnesis and physical examination, and confirmed through routine biochemical tests (complete blood cell count, glucose, insulin, lipids, lipoproteins, and thyroid function tests including TSH, free-T4, and liver and kidney function tests). Individuals younger than 18, with chronic diseases, systemic infections, or digestive-absorption disorders, smokers, alcohol consumers, regular drug users, and individuals using any food supplements were excluded from the study, in addition to pregnant, breastfeeding, and menopausal women. Informed consent forms were signed by all participants. Approval for the research was obtained from the Karadeniz Technical University (KTU) Faculty of Medicine Scientific Research Ethics Committee, Turkey (submission number 2020/54, approval date 9 March 2020), and the study was conducted in accordance with the principles of the Declaration of Helsinki.

### 2.2. Anthropometric Measurements

Anthropometric measurements were performed before the OFTT. The participants were weighed in bare feet, after overnight fasting, and in the morning. Heights was measured using a standard method. Body mass index (BMI) values were calculated as body weight in kilograms divided by height squared (kg/m^2^). Waist circumference was measured at the midpoint between the lowest rib and the iliac crest, and parallel to the floor. Hip circumference was measured at the level of the greater trochanter. Systolic blood pressure (SBP) and diastolic blood pressure (DBP) were measured after the participant had been in a sitting position for at least 10 min.

### 2.3. Dietary Assessments

Dietary consumption data were obtained from participants’ individual food records over three consecutive days, including two weekdays and one weekend day. Dietitians explained how these records should be kept. Participants were asked to record foods and beverages as they were consumed, paying particular attention to factors such as the name of the food, the amount consumed, its nutrient content, meal times, cooking methods, and the brand names of commercial products. The acceptable macronutrient distribution range (AMDR) represents the range of the percentages of daily energy obtained from each of the three macronutrients, including carbohydrate, fat, and protein [26]. AMDR values for daily energy intake for adults are 45–60% for carbohydrate, 20–35% for fat, and 10–20% for protein [27,28]. In the present study, the diet that the participants consumed without any restrictions in line with their routine eating habits was defined as the habitual diet (HD), and the diet prepared according to the AMDR recommended by the dietary guideline [27] was defined as the standard diet (SD). The standard diet was prepared under the supervision of a dietitian, taking into account the individuals’ daily energy and nutrient needs. The diet was created to be balanced in terms of food groups, paying particular attention to the sources of energy, carbohydrate, protein, and fat. The diets were explained to the participants, and food consumption records were again obtained for three consecutive days, covering the standard diet period. Daily energy and macronutrient intake data derived from the food records for the habitual and standard diets were calculated using the Nutrition Information System software (BeBIS, full version 9).

### 2.4. Oral Fat Tolerance Test

An OFTT based on the suggestions of the expert panel [11,12] was applied to each participant. The amounts of macronutrients in a single OFTT meal are those (25 g of carbohydrate, 10 g of protein, and 75 g of fat) recommended by the expert panel [11,12] and were also previously applied by our research group [21,29]. The OFTT meal consisted of a soup, which was composed of 100 g of regular whole milk yogurt, 7 g of rice flour, 200 mL of water, and 75 g butter, and two slices (40 g) of whole wheat bread, and was consumed in 20 min. Same brand commercial products were used to ensure standardization for the test meals. During the OFTTs, the participants were allowed to continue their daily activities unaltered, and were asked to avoid excessive exercise or bed-rest. They were also instructed not to consume anything else, except for water. The simplified procedure recommends that the TG level measured at the 4th h after the OFTT meal be regarded as the representative postprandial TG level measurement time, and this will facilitate the use of OFTT for clinical purposes [11,13,14,16]. In line with its speed, ease of application, least invasiveness and general applications in the literature, blood samples collected at fasting and 4 h after consumption of the OFTT meal were evaluated in this study. Delta TG values were calculated from TG concentrations measured at fasting and at the 4th h of OFTT.

### 2.5. Study Design

**Figure 1:**
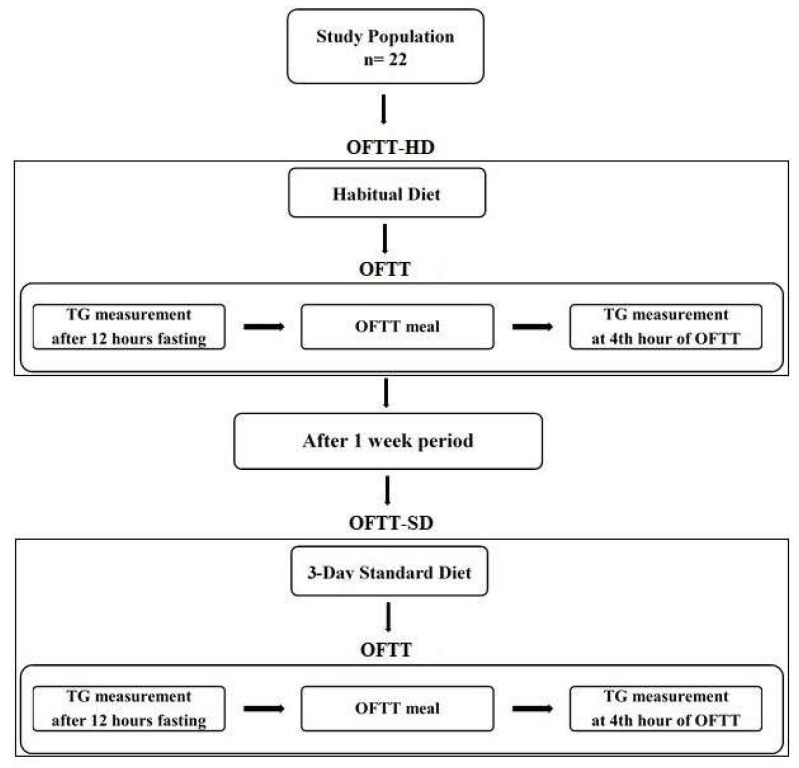
Schematic presentation of the experimental procedure.

A schematic representation of the experimental procedure is shown in Figure 1. Participants underwent two consecutive OFTTs. Once 3-day habitual diet food consumption records had been collected from each participant, the first OFTT was applied on the morning of the fourth day (OFTT-HD). A one-week wash-out period was allowed, after which each volunteer then consumed a 3-day standard diet. The same OFTT meal and procedure were administered to each individual. The second OFTT was applied on the following morning after the 3-day standard diet (OFTT-SD).

### 2.6. Biochemical Tests

Serum glucose, TG, total cholesterol (TC), low density lipoprotein-cholesterol (LDL-C), and high-density lipoprotein-cholesterol (HDL-C) concentrations were measured enzymatically using a Beckman Coulter AU 5800 series clinical chemistry analyzer (Shizuoka, Japan). Serum insulin concentrations were measured on an IMMULITE 2000 XPi immunoassay system (Siemens, Munich, Germany). The original reagents of the analyzers were used to measure the concentrations of variables. Biochemical variables were quantified in the Medical Biochemistry Laboratory of the KTU Farabi Hospital following the completion of daily quality control procedures. The homeostasis model assessment of insulin resistance (HOMA-IR) was calculated using the formula [fasting serum insulin (µU/mL) × fasting serum glucose (mg/dL)/405].

### 2.7. Statistical Analyses

The distribution of variables was assessed using the Shapiro–Wilk test. The data were expressed as mean and standard deviation (mean ± SD) for normally distributed variables. Non-normally distributed data were expressed as median with interquartile range (Q1–Q3) values. The paired samples *t*-test was used to compare normally distributed variables. Pairwise comparisons were performed using the Wilcoxon test in case of non-normal distribution. The McNemar test was used to compare proportions. The minimum number of individuals to be included in the study group was computed using the G*Power 3.1.9.7 software (Heinrich-Heine Universitat, Dusseldorf, Germany). A sample size of 17 with 80% power and a 5% type I error level was determined with the effect size calculated using the triglyceride mean and standard deviation values in the literature [30]. *p* values < 0.05 were considered statistically significant. Statistical analyses were performed on the SPSS version 23.0 software (SPSS, Inc., Chicago, IL, USA).

## 3. Results

### 3.1. Findings concerning the Macronutrient Content of Habitual and Standard Diets

The mean values for the intake of macronutrients as a percentage of the daily energy intake from the food records for the habitual and standard diets are presented in Table 1. The amounts of energy were not statistically significantly different (*p* = 0.913). The percentage of carbohydrate in the habitual diet (43%) was significantly lower than in the standard diet (49%) (*p* = 0.001), while the percentage of fat (38%) was significantly higher (*p* = 0.001). The standard diet increased the mean carbohydrate percentage by 15 points, and reduced the fat percentage by 14 points, according to the habitual diet. However, no significant change was observed in protein percentages (*p* = 0.823). Fiber intake increased by 80% with the standard diet. There was no change for the amount of cholesterol intake between the diets.

### 3.2. The Effects of the Standard Diet on Anthropometric and Biochemical Variables

The data for the anthropometric variables of body weight, BMI, waist circumference, hip circumference, SBP, and DBP are given in Table 2. No change in anthropometric data was observed with the standard diet. No significant difference was found in fasting glucose, insulin, HOMA-IR, and lipid levels following the standard diet either (Table 2).

### 3.3. The Effects of the Standard Diet on OFTT Variables

The median fasting and 4th h TG levels and delta TG values for the OFTT-HD and OFTT-SD are presented in Table 3. Fourth hour TG concentrations were significantly lower after the standard diet (*p* = 0.038). A decrease of 12.8% was observed compared to OFTT-HD. Although a 10% decrease in fasting TG concentrations and a 22.7% decrease in delta TG values were determined following the standard diet, these were not statistically significant.

The OFTT may be recommend for individuals with fasting TG concentrations of 89–180 mg/dL based on the suggestions of the expert panel. Therefore, in order to evaluate the effects of the standard diet on OFTT variables in individuals with fasting TG concentrations of 89–180 mg/dL or lower than 89 mg/dL, the study population was classified on the basis of fasting TG concentrations as follows: fasting TG < 89 mg/dL (n = 11, 6 female and 5 male) and fasting TG = 89–180 mg/dL (n = 11, 5 female and 6 male) (Table 4). The standard diet significantly reduced fasting and 4th h TG concentrations and delta TG values by 19.1%, 13.45%, and 28.7%, respectively, in individuals with fasting TG concentrations between 89 and 180 mg/dL (Table 4). However, these OFTT variables did not vary significantly in individuals with fasting TG levels < 89 mg/dL (Table 4).

**Figure 2:**
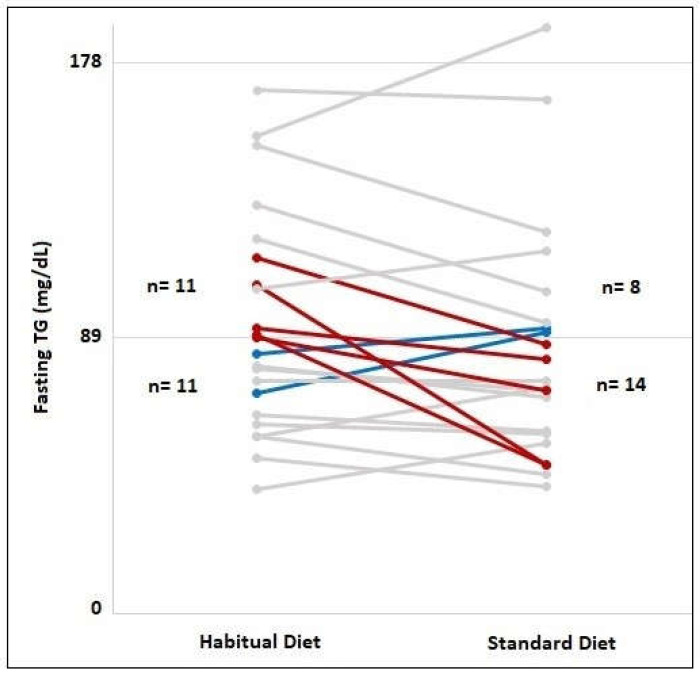
Frequency of individuals with fasting TG concentrations lower than 89 mg/dL and fasting TG concentrations between 89–180 mg/dL in habitual diet and 3-day standard diet consumption. The red lines indicate individuals with fasting TG concentrations between 89 and 180 mg/dL switched to TG concentrations below 89 mg/dL after the standard diet consumption. The blue lines indicate individuals with fasting TG concentrations below 89 mg/dL switched to TG concentrations between 89–180 mg/dL after the standard diet consumption. The gray lines indicate individuals whose fasting TG group did not change after the standard diet.

In order to compare the proportions of individuals with fasting TG concentrations of 89–180 mg/dL for habitual diet and standard diet, a McNemar test was performed. The number of individuals with a fasting TG value of 89–180 mg/dL, namely, 11 (50%), decreased to 8 (36.4%) following the 3-day standard diet (Figure 2, *p* = 0.453). After the standard diet, 5 (45.5%) of the 11 individuals with fasting TG values of 89–180 mg/dL had fasting TG values of <89 mg/dL (Figure 2). On the other hand, 2 (18%) of the 11 individuals with a fasting TG value of <89 mg/dL had a fasting TG value of 89–180 mg/dL after the standard diet (Figure 2).

The effects of the standard diet on OFTT variables according to gender differences were also assessed (Table 5). Decreases of 10.9%, 14.7%, and 26% were observed in fasting and 4th h TG levels and delta TG values, respectively, with the standard diet in females. However, no statistically significant alterations were found in these variables in males.

## 4. Discussion

The current study was designed to investigate whether a 3-day standard diet is required prior to the OFTT by evaluating the effects of this short-term diet on OFTT results. One of the main findings of the present study was a difference between the habitual diet and standard diet in terms of macronutrient composition, especially carbohydrate and fat percentages. Other important findings of this study were that the 3-day standard diet prior to the OFTT modulated the test results by reducing fasting and 4th h TG concentrations and delta TG values.

There is considerable evidence that the amount and type of carbohydrates, fat amount, fatty acid composition, and fiber amount taken with the diet affect plasma TG concentrations [24,25,31]. In previous studies, an intensive 3-day dietary manipulation with isoenergetic high-fat and high-carbohydrate diets has been used to investigate marked effects on both fasting and postprandial TG concentrations [30,32,33,34]. In the present study, we applied the standard diet for 3 days, considering that it would facilitate the period of adherence of the individuals to the diet, be sufficient in terms of the serum TG levels response, and facilitate its application in a clinical setting. In general, it has been observed that replacing dietary fat with excess carbohydrate increases plasma fasting TG levels, and that replacing carbohydrate with excess fat reduces plasma fasting TG levels [30,32,33,34]. The macronutrient composition of the diet can affect fasting and postprandial lipid and lipoprotein concentrations through different mechanisms that modulate their synthesis or catabolism [35,36]. Diet-induced differences in VLDL-TG synthesis and secretion, and the VLDL-TG clearance rate may contribute to changes in TG concentrations [35]. Our findings may therefore be due to reduced VLDL-TG synthesis and secretion, enhancement of hepatic fatty acid oxidation, and a rise in the VLDL-TG clearance rate following the standard diet. In addition, the lower postprandial lipemic response after the standard diet may be attributable to an increased TG clearance rate and a decreased fasting TG pool, which may reduce competition for lipoprotein lipase when newly synthesized chylomicrons appear after the OFTT.

The expert panel recommended that it would be more beneficial to evaluate individuals with fasting TG concentrations between 89 and 180 mg/dL using the OFTT for the diagnosis of postprandial lipemia [11]. Additionally, individuals with fasting TG concentrations <89 mg/dL do not generally exhibit an abnormal TG response to the OFTT and do not benefit from OFTT for the diagnosis of postprandial lipemia [11]. In line with these recommendations, we evaluated the effects of habitual and standard diets on OFTT results in two groups established on the basis of fasting TG concentrations. Our findings support these recommendations. The effects of the standard diet on OFTT results was particularly pronounced in individuals with fasting TG concentrations of 89–180 mg/dL. Individuals with fasting TG concentrations between 89 and 180 mg/dL can switch to TG concentrations below 89 mg/dL, which is not a recommended concentration for the administration of the OFTT, with the consumption of a 3-day standard diet prior to undergoing the OFTT. This is particularly important for individuals with fasting TG concentrations bordering on OFTT administration, since their fasting TG concentrations can change to values that are not recommended for OFTT administration following the standard diet, and the OFTT might be applied to individuals who should not be administered. Because subjects with fasting TG concentrations > 180 mg/dL have been predicted to exhibit an abnormal response at the 4th h (> 220 mg/dL) [11], they were excluded from the present study. Moreover, the standard diet reduced the 4th h TG concentrations of the OFTT by approximately 13%. This is important for the diagnosis of postprandial lipemia because a standard diet can prevent false positive results. Furthermore, three individuals in the study population had postprandial TG concentrations over 220 mg/dL. However, the number of individuals was small, and it was not included in the evaluation. It may be suggested that a 3-day standard diet is important both for individuals with borderline fasting TG concentrations recommended for test applicability and for individuals with a borderline positive response for postprandial lipemia. Thus, the decrease in TG concentration with the standard diet may prevent the test being applied to individuals who do not require it, and false negative or positive diagnosis.

Since gender is one of the important factors affecting the postprandial TG response, we also evaluated the effects of the standard diet on OFTT results in both females and males. This short-term diet was more effective in females than males. The findings from this study are consistent with our previous study, in which we showed that the postprandial lipemic response was lower in females than males [21]. Because the postprandial lipemic response is lower in females than in males, the recommended TG concentrations for OFTT administration should be re-evaluated on the basis of gender.

Fasting TC, LDL-C, and HDL-C levels did not change with the 3-day standard diet in our study. Plausible explanations for this observation may include the fact that the amount of cholesterol consumed in the diet remained unchanged, and that the duration of the diet containing acceptable macronutrient percentages recommended for healthy eating was not sufficiently long to alter plasma cholesterol concentrations. These observations are consistent with some results from previous short-term dietary interventions. Although studies in which the TC amount does not change after 3-day high-fat or high-carbohydrate diets are in the majority, the levels of LDL-C and HDL-C are contradictory [32,33,34]. The anthropometric variables also exhibited no change with the short-term standard diet in this study. This may be attributable to the standard diet being isocaloric with the individual’s habitual diet and being prepared in the form of a diet containing recommended AMDR, and not for the purpose of reducing or increasing body weight.

This study is limited in terms of extrapolating the findings to the general population due to the relatively small sample size, and therefore its findings should be confirmed in larger cohorts in future studies. Thus, subgroup analyses based on fasting TG and gender differences would also be carried out in larger sample sizes. The other limitation of this study is that it contained only three individuals with an abnormal postprandial response. This restricts our ability to evaluate the bias of the effects of the 3-day standard diet for the diagnosis of postprandial lipemia. The sample size of subjects having abnormal postprandial response could be increased in future studies. Moreover, the fact that the first OFTT could have a priming effect on the second OFTT may also be considered as a possible limitation.

## 5. Conclusions

It was concluded that a 3-day standard diet applied prior to the OFTT could modulate plasma fasting TG concentrations and the postprandial TG response. This effect was particularly pronounced in individuals with fasting TG concentrations between 89 and 180 mg/dL, the recommended range for the OFTT. The short-term standard diet intake prior to the OFTT may be useful in preventing unnecessary administration of the OFTT and in correctly identifying individuals who should receive the test. It may therefore be suggested that the application of a short-term standard diet before the OFTT may provide an improvement in terms of preanalytical errors and OFTT results. As a result, more standardized OFTT protocols, including prior to the test and testing period, are needed to assess the postprandial lipemic response.

## Figures and Tables

**Table 1:** Macronutrient values of the daily energy from the habitual diet and standard diet in healthy adults.

Variables	Habitual Diet	Standard Diet	Percent Change ^a^	*p*
Energy, kcal/day	1342 ± 314	1349 ± 316	3.79 ± 25.3	0.913
Carbohydrate, E%	43 ± 6.53	49 ± 3.23	15.0 ± 17.7	0.001
Fat, E%	38 ± 4.66	32 ± 3.43	−14.0 ± 12.4	0.001
Protein, E%	19 ± 3.26	19 ± 1.75	3.98 ± 20.5	0.823
Fiber, g	15 ± 6.10	24 ± 9.22	80 ± 93	0.001
Cholesterol, mg	272 ± 99	281 ± 79	15.4 ± 45.5	0.713

Abbreviations: E%, percentage of the daily energy intake; ^a^, percent change according to the habitual diet. Values are given as mean ± standard deviation. The paired samples *t*-test was used for statistical analysis.

**Table 2:** Anthropometric and biochemical variables after habitual diet and standard diet in healthy adults.

Variables	Habitual Diet	Standard Diet	*p*
Body weight, kg	71.1 ± 11	70.7 ± 10.7	0.083
BMI, kg/m^2^	24.7 ± 2.61	24.6 ± 2.55	0.121
Waist circumference, cm	84.4 ± 10.1	84.5 ± 9.95	0.833
Hip circumference, cm	99.1 ± 6.62	99 ± 6.72	0.427
SBP, mm/Hg	112 ± 13.1	111 ± 12.5	0.329
DBP, mm/Hg	75.5 ± 8.58	74.6 ± 7.39	0.162
Glucose, mg/dL	92 (86.8–95.5)	92 (86.8–97.3)	0.810 *
Insulin, mIU/L	6.22 (4.90–10.2)	6.19 (4.02–9.21)	0.122 *
HOMA-IR	1.43 (1.02–2.29)	1.40 (0.79–2.06)	0.131 *
TG, mg/dL	86 (63–116)	74 (57–96)	0.118 *
TC, mg/dL	204 ± 33.3	203 ± 37.3	0.681
LDL-C, mg/dL	133 ± 29	132 ± 31	0.754
HDL-C, mg/dL	52 (46–57)	52 (46–56)	0.519 *

Abbreviations: BMI, body mass index; SBP, systolic blood pressure; DBP, diastolic blood pressure; HOMA-IR, homeostatic model assessment of insulin resistance; TG, triglyceride; TC, total cholesterol; LDL-C, low-density lipoprotein cholesterol; HDL-C, high-density lipoprotein cholesterol. * Values are given as median with IQR (Q1–Q3) and the Wilcoxon test was used for statistical analysis. The remaining variables are given as mean ± standard deviation and the paired samples *t*-test was used for statistical analysis.

**Table 3:** Fasting TG, 4th h TG, and delta TG values of OFTT after habitual diet and standard diet in healthy adults.

Variables	OFTT-HD	OFTT-SD	Percent Change ^a^	*p*
TG-fasting, mg/dL	86 (63–116)	74 (57–96)	−10.0 (−21.1–10)	0.118
TG-4th h, mg/dL	148 (103–196)	124 (83–149)	−12.8 (−26.5–7.54)	0.038
Delta TG	54 (36–103)	44 (22–73)	−22.7 (−54–27)	0.058

Abbreviations: TG, triglyceride; OFTT, oral fat tolerance test; OFTT-HD, oral fat tolerance test after habitual diet; OFTT-SD, oral fat tolerance test after standard diet; ^a^, percent change according to the OFTT-HD. The variables are given as median with IQR (Q1–Q3) and the Wilcoxon test was used for comparisons.

**Table 4:** Fasting TG, 4th h TG, and delta TG values of OFTT after habitual diet and standard diet according to fasting TG concentration groups in healthy adults.

	TG < 89 mg/dL (n = 11)				TG = 89–180 mg/dL (n = 11)			
Variables	OFTT-HD	OFTT-SD	Percent Change ^a^ TG < 89	*p* *	OFTT-HD	OFTT-SD	Percent Change ^a^ TG = 89–180	*p* **
TG-fasting, mg/dL	64 (57–79)	70 (55–75)	−4.9 (−12.5–28.1)	0.721	115 (92–151)	94 (72–123)	−19.1 (−23.5–1.78)	0.062
TG-4th h, mg/dL	103 (94–146)	108 (63–140)	−12.2 (−19.4–22.9)	0.533	194 (154–236)	146 (120–228)	−13.45 (−38.1–0.80)	0.018
Delta TG	38 (22–71)	33 (18–55)	−22.7 (−50–52)	0.534	93 (49–112)	50 (24–98)	−28.7 (−71.6–13.7)	0.047

Abbreviations: TG, triglyceride; OFTT, oral fat tolerance test; OFTT-HD, oral fat tolerance test after habitual diet; OFTT-SD, oral fat tolerance test after standard diet; ^a^, percent change according to the OFTT-HD. The variables are given as median with IQR (Q1–Q3) and the Wilcoxon test was used for comparisons. * *p* values for TG < 89 mg/dL, ** *p* values for TG = 89–180 mg/dL.

**Table 5:** Fasting TG, 4th h TG, and delta TG values of OFTT after habitual diet and standard diet according to gender differences in healthy adults.

	Female				Male			
	(n = 11)				(n = 11)			
Variables	OFTT-HD	OFTT-SD	Percent Change ^a^ Female	*p* *	OFTT-HD	OFTT-SD	Percent Change ^a^ Male	*p* **
TG-fasting, mg/dL	79 (61–106)	72 (55–82)	−10.9 (−21.2–4.92)	0.028	90 (71–151)	91 (70–123)	−1.78 (−21.1–22.7)	0.756
TG-4th h, mg/dL	146 (102–194)	108 (67–148)	−14.7 (−28.1–5.71)	0.041	154 (103–236)	129 (123–228)	−3.39 (−16.8–9.65)	0.374
Delta TG	54 (38–82)	43 (19–52)	−26 (−54–13)	0.142	67 (31–123)	52 (23–98)	−18 (−60–45)	0.169

Abbreviations: TG, triglyceride; OFTT, oral fat tolerance test; OFTT-HD, oral fat tolerance test after habitual diet; OFTT-SD, oral fat tolerance test after standard diet; ^a^, percent change according to the OFTT-HD. The variables are given as median with IQR (Q1–Q3) and the Wilcoxon test was used for comparisons. * *p* values for female, ** *p* values for male.

## Data Availability

The datasets generated during and/or analyzed during the current study are available from the corresponding author on reasonable request. The data are not publicly available due to privacy.
